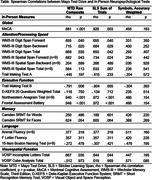# Usability of a Self‐Administered Cognitive Assessment in Patients with Progressive Supranuclear Palsy and Correlation with Traditional Neuropsychological Tests: A Mayo Test Drive Pilot Study

**DOI:** 10.1002/alz.085992

**Published:** 2025-01-03

**Authors:** Elizabeth A. Boots, Megan J. Broeren, Farwa Ali, Heather M. Clark, Julie A.G. Stierwalt, Hugo Botha, Yehkyoung C. Stephens, Keith A. Josephs, Jennifer L. Whitwell, Mary M. Machulda, Nikki H. Stricker

**Affiliations:** ^1^ Mayo Clinic, Rochester, MN USA

## Abstract

**Background:**

Self‐administered cognitive assessments demonstrate usability and ability to detect cognitive decline in Alzheimer’s disease, but usability in other neurodegenerative diseases is understudied. We investigated whether Mayo Test Drive (MTD), a self‐administered multi‐device compatible cognitive assessment platform, demonstrates usability and correlation with traditional neuropsychological tests in a pilot study of individuals with progressive supranuclear palsy (PSP).

**Method:**

Eleven individuals with PSP (mean age = 69.6±7.2 years; mean education = 15.3±2.7 years; 72.7% male; 90.9% white; PSP Rating Scale [PSPRS] = 32.4±15.5) enrolled in a Neurodegenerative Research Group study at Mayo Clinic participated. Participants completed neurological evaluation, traditional neuropsychological assessment, in‐person self‐administered MTD comprised of the Stricker Learning Span and Symbols Test, and a MTD usability questionnaire with quantitative and qualitative questions. Participants received diagnoses of PSP clinical variants per Movement Disorders Society‐PSP criteria. Seven participants had PSP‐Richardson, two had PSP‐postural instability, and two had PSP‐parkinsonism. We used descriptive analyses to examine usability questions, qualitative analyses for free text responses, and Spearman correlations to analyze associations between MTD and traditional tests of global cognition and five cognitive domains.

**Result:**

Ten of eleven patients completed MTD (average completion time [minutes] = 22.2±6.5). Study staff terminated one participant’s session early due to an appointment conflict; we excluded this participant from analyses. Six participants completed MTD with tablets and four used smartphones. Participants reported high ease completing MTD (9.00±1.76 on 0‐10 scale) and comfortability using their devices (9.40±1.58). All participants reported they could complete MTD independently, with one participant indicating need for assistance with hyperlinks and three reporting need for assistance with QR codes. Qualitative data suggested three participants had motoric difficulty using the tablets; these individuals had the greatest PSPRS symptom severity, although MTD performance was not correlated with the PSPRS (rho = ‐.122; p>.05). MTD performance significantly correlated with the MoCA (rho = 0.88) and tests of attention, executive, and visuospatial function (Table).

**Conclusion:**

In a MTD usability pilot for participants with PSP, participants reported high ease and comfort completing MTD independently; those with greater motor symptoms had more difficulty navigating technology. Additionally, performances on MTD were correlated with the MoCA and other in‐person assessments, demonstrating convergent validity.